# Editorial: Digitizing plant breeding: big data meets genetic mapping

**DOI:** 10.3389/fpls.2023.1278580

**Published:** 2023-10-04

**Authors:** Kyung Do Kim, Justin Vaughn, Changsoo Kim

**Affiliations:** ^1^ Department of Bioscience and Bioinformatics, Myongji University, Yongin, Republic of Korea; ^2^ Agricultural Research Service, United States Department of Agriculture, Washington, DC, United States; ^3^ Department of Crop Science, Chungnam National University, Daejeon, Republic of Korea

**Keywords:** big data, genomics, transcriptomics, QTL mapping, plant breeding, digitizing, bioinformatics

Advancements in genomics and high-throughput technologies have revolutionized plant breeding, providing researchers with an abundance of big data encompassing various -omic experiments for both model and commodity crops. However, despite the wealth of publicly available data, many researchers in the field of quantitative trait locus (QTL) mapping continue to rely primarily on traditional genetic associations, failing to harness the full potential of this vast information for further locus refinement and gene discovery. Researchers should be able to gather and use all relevant, publicly available data related to their loci of interest and specific traits. By doing so, they can unlock new opportunities for accelerating the breeding process and developing crop varieties with improved yields, disease resistance, and other desirable traits. This Research Topic on “*Digitizing Plant Breeding: Big Data Meets Genetic Mapping*” presents five research articles highlighting the need for a more coherent, searchable resource that can bridge the gap between big data and genetic mapping in plant breeding.

## 1 Genome database and bioinformatic pipelines

*Brassica oleracea* is a plant species that encompasses a wide range of vegetable crops, such as cabbage, broccoli, cauliflower, kale, and kohlrabi. Wang et al. developed the *B. oleracea* Genome Database (BoGDB), an integrative platform that consolidates genome, transcriptome, and metabolome data of different *B. oleracea* cultivars. It provides access to the genome sequences and annotations of seven *B. oleracea* cultivars with different morphologies, as well as the transcriptome and metabolome data of various tissues and organs. It also offers a variety of tools and modules for data analysis and visualization, such as gene search, heatmap, genome browser, BLAST, gene family search, transcription factor search, protein kinase search, flanking sequence finder, GO enrichment, KEGG enrichment, metabolic pathway, and variation analysis.

BoGDB enables users to explore and compare the genomic features and evolutionary dynamics of *B. oleracea* cultivars, as well as identify genes and pathways involved in important traits and functions. For example, the authors demonstrate the application of BoGDB by conducting an analysis of the ABC transporter gene family in *B. oleracea*, which is involved in plant defense and stress responses. The analysis reveals the phylogenetic relationships, gene structures, expression patterns, and functional roles of the ABC transporter genes in *B. oleracea*.

BoGDB is a timely and welcome addition to the existing databases of Brassicaceae species. It will facilitate the genomic research and molecular breeding of *B. oleracea* crops and stimulate further investigations into the mechanisms underlying the phenotypic diversity and genome evolution of *B. oleracea*. BoGDB is a valuable resource for researchers and breeders interested in *B. oleracea* genetics and genomics.

Exploiting the full potential of the genetic diversity available in rice germplasm collections is significant for the development of new and improved varieties of rice. Darwell et al. present a novel bioinformatics pipeline, RICEEXPLORER, that can integrate local and global genomic data to identify functional haplotypes associated with desirable traits, such as grain length. RICEEXPLORER can also perform various analyses to assess the diversity, linkage disequilibrium, phylogenetic, and population genomic relationships among rice accessions. The authors demonstrate the utility of RICEEXPLORER by applying it to a Thai rice resource (TRR) panel, consisting of elite cultivars and non-focal varieties, such as landraces. They show that RICEEXPLORER can uncover the hidden potential of non-focal varieties that harbor large grain size-associated haplotypes that are absent or rare in elite cultivars. They also suggest that RICEEXPLORER can be used for any agricultural focal species with appropriate genotype–phenotype data.

This article is an impressive example of how bioinformatics can facilitate and enhance crop breeding programs. The authors have developed a user-friendly and comprehensive pipeline that can generate valuable information for breeders and researchers. RICEEXPLORER can help identify novel sources of genetic variation, functional variants, and haplotypes that may improve crop performance and resilience. It can also provide insights into the evolutionary history and genomic architecture of rice and other crops. The authors have made their pipeline publicly available and provide detailed instructions on how to use it. RICEEXPLORER is a powerful tool that can advance the field of plant genomics and breeding.

## 2 GWAS and transcriptomics in tree species

Leaves exhibit very different photosynthetic efficiency in high versus low light. This discrepancy is driven by numerous ecological limits but tends to result in adaptive leaf shapes unique to a species. Heterophylly - i.e. variable leaf shape along a single plant - is clearly of ecological and agronomic interest since heterophyllic species seem to be adopting a more complex light harvesting regime. Still, an understanding of the specific adaptive nature of the trait remains hindered by sparse information about its genetic basis.

Zhu et al. have attempted to address this gap by performing a genome-wide association study (GWAS) using 860 *Populus euphrata* accessions. Tree species are ideal for GWAS since they generally exhibit very low linkage disequilibrium and thus can enable gene-level genetic resolution. In this study, the authors divide leaves into specific shape types. Within each category, they perform GWAS on individual classic metrics, like width, as well as the principal components of these combined metrics. Somewhat strikingly, the authors observe few overlapping peaks, even between similar leaf types. Most QTL appear to be of low effect and, thus, have borderline significant scores. The authors also attempt to aggregate trait metrics for individual plants across leaf types. As expected, associations are even weaker but suggests there is some degree of shared causal variation.

While the study represents a critical look into this complex phenotype, much future work is in store. Two extensions are of critical importance: 1) Long read sequencing could be easily applied to improve the decade-old reference used in the study. Indeed, many QTL appear to lie in unplaced scaffolds. Still further, researchers could assemble genomes from numerous key individuals and get a fuller representation of both single nucleotide *and* structural variation in a pangenomic context. 2) The natural population used in the study prevented replication and assumedly added a large environmental effect that cannot be accounted for. Additionally, the trees were not the same age. Some indication of the heritability of the trait and the variance explained by discovered QTL will go far in evaluating the importance of these results. Also, a more rigorous experimental design might allow critical aspects of the plasticity of this trait to be investigated as well. We look forward to further insights from this research.

Grapevine (*Vitis vinifera*) is a highly complex plant species with a rich genetic diversity that contributes to its wide range of flavors, aromas, and adaptability to diverse environments. Transcriptomic analysis plays a pivotal role in unraveling the genetic basis of grapevine development and response to environmental stimuli. Moretto et al. have presented a novel approach that not only sheds light on the grapevine transcriptome but also adheres to the principles of FAIR (Findable, Accessible, Interoperable, and Reusable) data sharing through the database “VESPUCCI”. In this study, the authors have developed the COMPASS GraphQL interface, a comprehensive pipeline for analyzing and visualizing grapevine transcriptomic data. This innovative approach incorporates the FAIR principles from the outset, ensuring that the generated data is findable, accessible, interoperable, and reusable. The current version presents improved features in terms of its normalization protocol, which accommodates different types of transcriptomic data. The following are some features and benefits provided by the current version:

### • Findability:

The COMPASS pipeline employs a standardized metadata schema, enabling efficient data discovery through robust indexing and search capabilities. This enhances the visibility of the dataset, facilitating its identification and utilization by the scientific community.

### • Accessibility:

The authors have made a concerted effort to ensure the accessibility of the transcriptomic data by implementing an open data policy. Through the use of publicly available repositories, such as the GrapeGen database, the data is freely accessible to researchers worldwide, promoting collaboration and knowledge exchange.

### • Interoperability:

COMPASS employs well-established data formats and ontologies, ensuring seamless interoperability with other transcriptomic datasets. This allows for the integration of data from multiple studies, facilitating comparative analyses and the identification of shared biological mechanisms.

### • Reusability:

By providing comprehensive documentation and standardized data formats, the authors have prioritized the reusability of their dataset. This not only enables the reproducibility of the study but also facilitates further investigations and hypothesis testing by the broader scientific community.

## 3 Comparative genomics of NLR resistance genes

Nucleotide-binding leucine-rich repeat (NLR) resistance genes play a vital role in plants’ defense against pathogens. The evolutionary history of NLR genes, particularly in non-model plant species like magnoliids, remains poorly understood. Wu et al. employed comparative genomics and phylogenetic analyses to explore the genomic repertoire of NLRs in various magnoliid species. The findings reveal a striking evolutionary trajectory characterized by the dramatic expansions of CNLs (Coiled-Coil NLRs) and the multiple losses of TNLs (Toll/Interleukin-1 Receptor-like NLRs) in magnoliids. The proliferation of CNLs shows that these genes provide an adaptive advantage, possibly because of evolutionary pressures imposed by various pathogenic risks. The loss of TNLs in magnoliids raises intriguing questions regarding the alternative mechanisms that may have evolved to compensate for this loss and maintain an effective defense response.

The knowledge gained from this study has practical implications for crop improvement strategies. By elucidating the evolutionary dynamics of NLR resistance genes, researchers can better understand the mechanisms of resistance and design targeted approaches to enhance plant defense against pathogens. This information can aid in the development of disease-resistant crop varieties, reduce reliance on chemical pesticides, and promote sustainable agriculture. The insights gained from this study pave the way for further investigations into the functional roles of the expanded CNLs and the compensatory mechanisms associated with TNL losses in magnoliids. The identification of specific NLR genes with potential agronomic importance and their functional characterization in crop species could revolutionize disease-resistance breeding programs. The identification of specific NLR genes with potential agronomic value and functional characterization in crop species could revolutionize disease-resistance breeding efforts.

## 4 Future trend of digitizing plant breeding

The future trajectory of breeding, characterized by the utilization of extensive big data (commonly referred to as digital breeding), is moving towards AI-driven breeding with minimal human intervention. This signifies that the ultimate objective of digital breeding is the impartial selection of desired plant characteristics, devoid of direct human involvement. To materialize this vision, researchers must systematically collect genotype and phenotype data from diverse populations, thus forming dependable models for specific trait.

The journey to digitize the breeding process is in its early stages, with researchers dedicated to enhancing digital breeding models and accumulating genetic data in various environmental contexts. It is our aspiration that the current Research Topic will serve as the cornerstone of digital breeding, facilitating future advancements in this field.

In summary, the success of digitizing plant breeding lies not only in generating vast amounts of data but also in effectively utilizing that data to drive targeted research and enhance crop improvement efforts. We believe that this Research Topic can offer valuable insights into the current challenges integrating big data with genetic mapping and can also present solutions towards a more unified, data-driven approach in plant breeding.

## Author contributions

KK: Writing – original draft, Writing – review & editing. JV: Writing – original draft, Writing – review & editing. CK: Writing – original draft, Writing – review & editing.

